# IL-4 Protects the Mitochondria Against TNFα and IFNγ Induced Insult During Clearance of Infection with *Citrobacter rodentium* and *Escherichia coli*

**DOI:** 10.1038/srep15434

**Published:** 2015-10-20

**Authors:** Arpan K. Maiti, Sinan Sharba, Nazanin Navabi, Huamei Forsman, Harvey R. Fernandez, Sara K. Lindén

**Affiliations:** 1 Department of Medical Biochemistry and Cell Biology, Sahlgrenska Academy, University of Gothenburg, Gothenburg, Sweden; 2 Department of Rheumatology and Inflammation Research, Sahlgrenska Academy, University of Gothenburg, Gothenburg, Sweden

## Abstract

*Citrobacter rodentium* is a murine pathogen that serves as a model for enteropathogenic *Escherichia coli. C. rodentium* infection reduced the quantity and activity of mitochondrial respiratory complexes I and IV, as well as phosphorylation capacity, mitochondrial transmembrane potential and ATP generation at day 10, 14 and 19 post infection. Cytokine mRNA quantification showed increased levels of IFN*γ*, TNF*α*, IL-4, IL-6, and IL-12 during infection. The effects of adding these cytokines, *C. rodentium* and *E. coli* were hence elucidated using an *in vitro* colonic mucosa. Both infection and TNF*α*, individually and combined with IFN*γ*, decreased complex I and IV enzyme levels and mitochondrial function. However, IL-4 reversed these effects, and IL-6 protected against loss of complex IV. Both *in vivo* and *in vitro*, the dysfunction appeared caused by nitric oxide-generation, and was alleviated by an antioxidant targeting mitochondria. IFN*γ* −/− mice, containing a similar pathogen burden but higher IL-4 and IL-6, displayed no loss of any of the four complexes. Thus, the cytokine environment appears to be a more important determinant of mitochondrial function than direct actions of the pathogen. As IFN*γ* and TNF*α* levels increase during clearance of infection, the concomitant increase in IL-4 and IL-6 protects mitochondrial function.

Infection with the attaching and effacing (A/E) murine pathogen *Citrobacter rodentium* is used as a model for studying the effects of other A/E pathogens that cause human diseases, such as enteropathogenic *E. coli* (EPEC) and enterohemorrhagic *E. coli* (EHEC)^1^,^2^,^3^. *C. rodentium* infection causes colitis characterised by crypt hyperplasia, goblet cell depletion and the presence of transmural inflammatory infiltrate^4^. In concert with these features, enhanced crypt epithelial cell death is also observed both in *C*. *rodentium* infected colon and in *E coli* infected human epithelial cells^1^,^5^,^6^,^7^.

Mitochondria play pivotal roles in cell function, providing most of the cell’s energy and participating in the Ca^2+^, redox and pH homeostasis^8^,^9^. Thus, major mitochondrial dysfunction is likely to make the cells more susceptible to factors leading to cell death. Several inter-related mitochondrial pathways regulate cell death processes, mainly by disrupting the mitochondrial respiratory chain resulting in a decrease in adenosine triphosphate (ATP) production; opening the mitochondrial permeability transition pore causing dissipation of membrane potential; release of cytochrome c; alteration of the cell’s redox status; and overproduction of reactive oxygen species^8^,^9^.

*C. rodentium* uses the same machinery as A/E *E. coli* to infect the host, attaching to the surface of intestinal epithelial cells through formation of a type III secretion system (T3SS)^1^,^2^,^3^. These bacteria use the T3SS to inject effector proteins, including the mitochondrial associated protein (Map) and several virulence factors like EspF, EspG and EspH into host cells^3^,^10^,^11^,^12^. EspF and Map are known to translocate into the host mitochondria and are involved in the disruption of normal cellular physiological functions^11^,^13^,^14^,^15^. Previous studies have shown that in murine *C. rodentium* infection, EspF targets mitochondria to initiate the host cell death pathway by alteration of membrane potential and release of cytochrome *c* into the cytoplasm^11^,^14^,^16^. Six days after *C. rodentium* infection, Map was found co-localised with host mitochondria, concurrent with a decrease in immunohistological staining for succinate dehydrogenase (SDH, complex II)^13^. However, the effects of *C. rodentium* on the other mitochondrial respiratory complexes involved in the electron transport chain, complexes I, III and IV, have not been examined.

Direct attachment of bacteria or injection of bacterial effector proteins can thus cause mitochondrial dysfunction of luminal epithelial cells^13^,^14^,^16^ , but mitochondrial pathway mediated cell death has also been observed in basal crypt epithelial cells, even though *C. rodentium* are rarely found at the bottom of the crypts^5^. This observation raises the possibility that cytokines upregulated during infection play a role in these responses, since cytokines influence mitochondria in other pathological conditions^17^.

The aim of the present study was to examine the status of mitochondrial enzymes and function during infection and clearance in the murine *C. rodentium* infection model, and delineate the role of the bacteria *per se* versus cytokines induced during different time points of the infection, using an *in vivo* like polarised *in vitro* epithelial mucosal surface that secretes a mucus layer^18^. We found mitochondrial dysfunction in the murine colonic epithelial cells following *C. rodentium* infection, in particular inhibition of complex I and IV of the mitochondrial respiratory chain, and loss of mitochondrial membrane phosphorylation capacity, membrane potential and ATP generation. The *in vitro* experiments indicated that the mechanism behind the mitochondrial dysfunction involved interferon gamma (IFN*γ*), tumour necrosis factor alpha (TNF*α*) and *C. rodentium* decreasing complex I and IV quantity and activity through activation of the nitric oxide (NO) pathway. IL-4, overexpressed only during the infection clearance phase, partially abrogates the mitochondrial dysfunction by reducing enhanced NO production, signifying the beneficial role IL-4 might play during infection clearance.

## Results

### Infection with *C. rodentium* induced colitis and cell death

**Figure 1:**
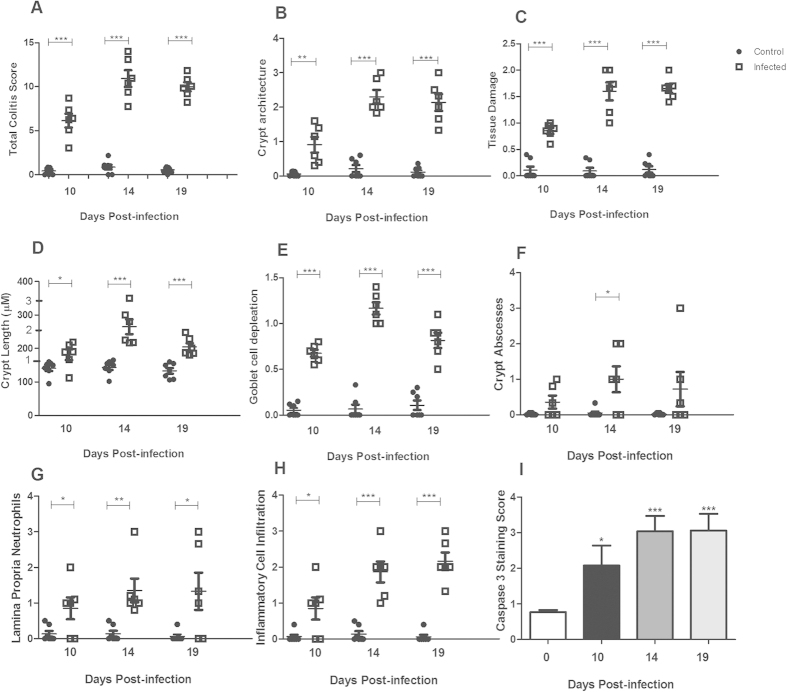
Colitis and caspase-3 staining during *C. rodentium* infection in the distal colon of WT mice. (**A**) The total colitis score represents the sum of the individual scores of the following parameters: (**B**) crypt architecture, (**C**) tissue damage, (**D**) crypt length (the numbers 1–3 on the y-axes indicates how the crypt lengths were translated to scores for the incorporation into the total colitis score), (**E**) goblet cell depletion, (**F**) crypt abscesses, (**G**) neutrophils in lamina propria and (**H**) inflammatory cell infiltration. Values are mean ± S.E.M (n = 6–7 mice). Statistics: unpaired t test, *P < 0.05, **P < 0.01, ***P < 0.001 compared to uninfected control. (**I**) Caspase-3 quantification. Statistics: ANOVA with Student Newman-Keuls Multiple Comparison post hoc test: *P < 0.05, **P < 0.01, ***P < 0.001. vs. control. The infection experiments were performed twice, and each time point contains results pooled from 4–9 mice.

We have previously shown that in *C. rodentium* infected C57BL/6 mice, the highest pathogen density in the feces is reached around day 10, then starts to decrease at day 14 and finally the infection is cleared (i.e. less than 100 CFU *C*. *rodentium*/g feces) around day 19^19^. We therefore focused on these three time points. Infection with *C*. *rodentium* produced features typical of colitis in wild type C57BL/6 (WT) mice (*P* < 0.001, Fig. 1A). On day 10, infected mice had mild overall colitis (Fig. 1A), but marked goblet cell depletion (Fig. 1E). On day 14 and 19 post-infection, there was an increase in crypt length, presence of neutrophils in the lamina propria and goblet cell depletion (Fig. 1B–H). In line with previous studies demonstrating cell death and sloughing of cells during *C. rodentium* infection^19^, the presence of the active cleaved form of caspase-3, indicative of apoptosis, increased in both the luminal surface and in the crypts (Fig. 1I). The caspase-9-caspase-3 cascade is activated by pro-apoptotic molecules such as cytochrome c released from mitochondria^20^,^21^.

### Loss of immunohistochemical (IHC) staining intensity for mitochondrial respiratory enzyme complex I, II and IV in infected WT mice

**Figure 2:**
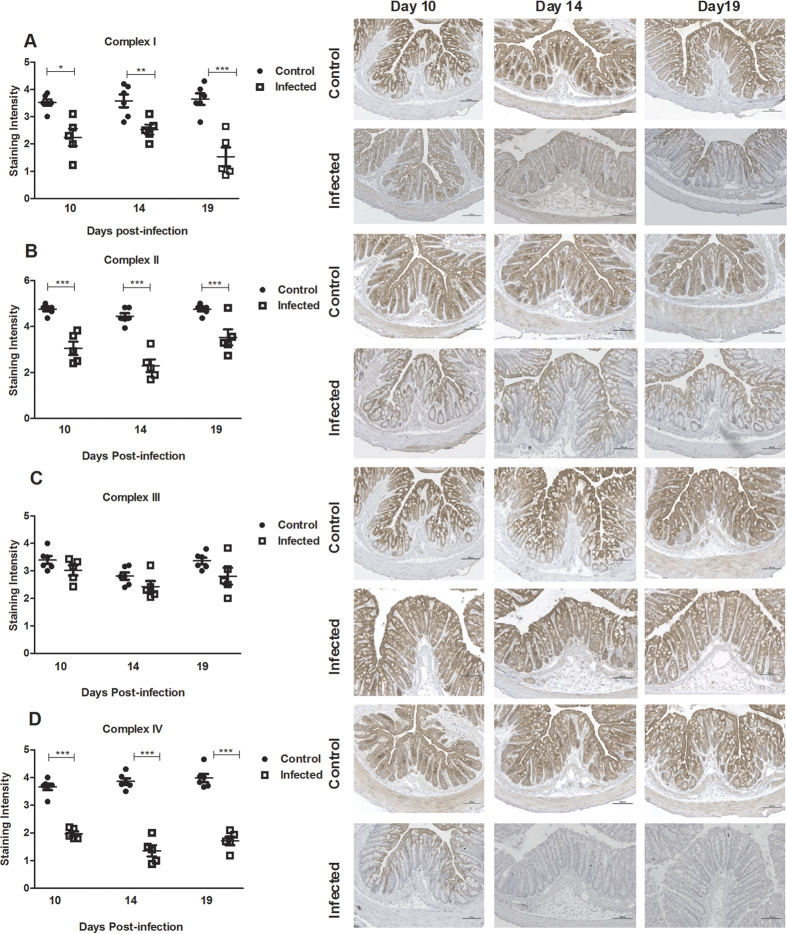
Tissue localization and semi-quantification of the mitochondrial respiratory enzyme complexes in the murine distal colon during *C. rodentium* infection. Immunohistochemical staining using antibodies for (**A**) MTND6 (complex I) (**B**) SDHA (complex-II) (**C**) CYC1 (complex-III) (**D**) CCO-VIc (complex-IV). Statistics: unpaired t test. *P < 0.05, **P < 0.01, ***P < 0.001 vs. control, n = 5–6 mice/group. The infection experiments were performed twice, and each time point contain results pooled from 5–6 mice. Scale bar 100 μm, magnification × 200.

Electron microscopy has previously shown that the mitochondria are located uniformly in non-goblet cells of the colon^22^. In the full goblet cells, the mucin granulae displaces most of the mitochondria to the rim of the cells, and evacuation of the mucin droplets discloses a rich content of mitochondria spread throughout the cytoplasm^22^. In line with this, the IHC staining patterns of all four complexes (complex I-IV) were relatively uniform in the majority of the epithelial surface cells, whereas the full goblet cells displayed pale areas where the mucin granulae are present (Fig. 2). During infection (day 10, 14 and 19) with *C. rodentium*, the intensity of the immunohistochemical staining for complex I, II and IV decreased in the epithelial cells (*P* < 0.05, Fig. 2A,B and D). However, no loss of staining intensity for complex-III was observed (Fig. 2C).

### Infection with C. rodentium caused dysfunction of mitochondrial respiratory enzyme complexes I and IV in infected WT mice

**Figure 3:**
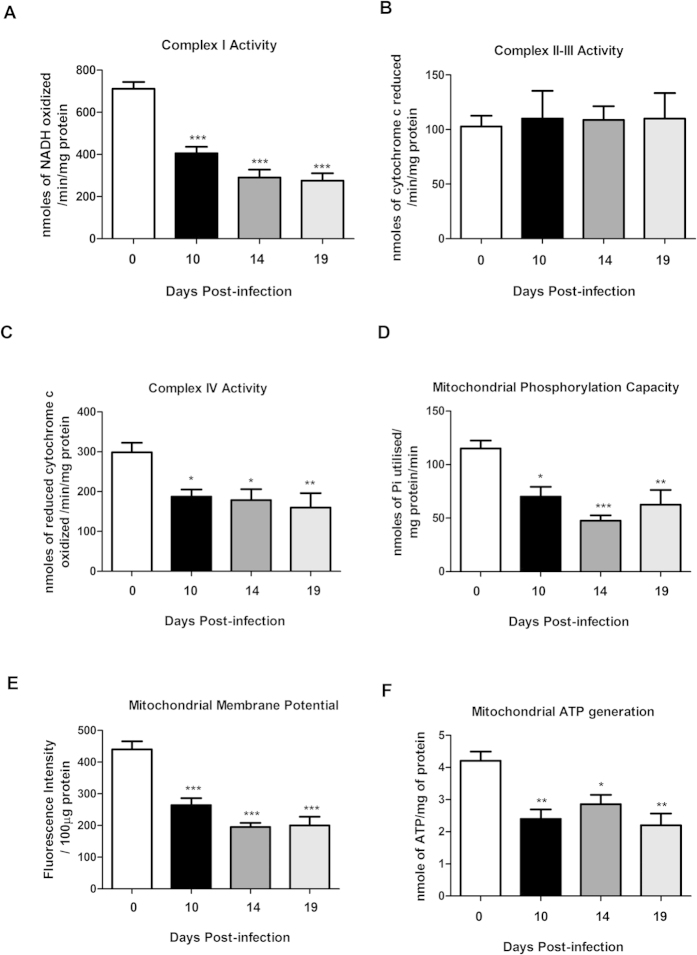
Mitochondrial function in the murine distal colon after *C. rodentium* infection. (**A**) complex I activity (**B**) complex II-III activity (**C**) complex IV activity (**D**) mitochondrial phosphorylation capacity (**E**) mitochondrial Membrane potential (**F**) mitochondrial ATP generation. Values are mean ± S.E.M. Statistics: ANOVA with Student Newman-Keuls Multiple Comparison post hoc test: *P < 0.05, **P < 0.01, ***P < 0.001 vs. control. (n = 4–9 mice/group). The infection experiments were performed twice, and each time point contains results pooled from 4–9 mice.

Next, we investigated if the decrease of staining intensity of mitochondrial complexes reflected their activity. Complex-I activity decreased by 43% during the mid-infection time point at 10 days post-infection, further decreased by day 14 (−59%) and remained low through to day 19 (−61%, *P* < 0.001, Fig. 3A). Similarly, complex-IV activity was reduced during infection (day 10: −37%, day 14: −40%, day 19: −46%, *P* < 0.05, Fig. 3C). No loss of enzymatic activity was observed at any time points for complex-II-III activity (Fig. 3B). In addition to the unchanged complex III protein levels and activity, we did not detect any loss in citrate synthase activity with infection (p = 0.6; control mice; 1.146 ± 0.10 U/mg protein, infected mice day 10; 1.046 ± 0.12 U/mg protein, infected mice day 14; 1.02 ± 0.026 U/mg protein). Together, this indicates that the amount of mitochondria do not decrease^23^, but that a loss of mitochondrial functionality occurs.

### *C. rodentium* infection caused a reduction of phosphorylation capacity, mitochondrial transmembrane potential and ATP generation in infected WT mice

The mitochondrial phosphorylation capacity was decreased by 60% at day 14 post infection, and by 45% at day 19 (*P* < 0.01, Fig. 3D). The mitochondrial transmembrane potential was decreased by at least 40% at all time points of infection (*P* < 0.001, Fig. 3E). Thus, infection impaired most factors important for mitochondrial respiration, and indeed, the ATP generation ability also decreased by up to 47% (*P* < 0.01, Fig. 3F).

### Both pro- and anti-inflammatory cytokines are expressed *in vivo* during *C. rodentium* infection

In order to identify the cytokines that may be impacting mitochondrial function we used an RT-PCR array of Th1/Th2 related genes to examine how the cytokine profile differed between day 10, 14 and 19 post *C. rodentium* infection. IFN*γ* and IL-12 mRNA were upregulated at all time points, whereas TNF*α* and IL-4 upregulation started at day 14 and IL-6 mRNA only increased at day 19 post infection (Table 1). The increased levels of TNF*α*, IFN*γ* and IL-12 are in line with a previous study using different time points^24^, and the increased levels of IL-4, TNFα and IFN*γ* at day 19 post infection was confirmed using individual RT-PCRs (fold increase mean [range] IL-4 5.9 [2.4–12.6], TNFα 5.3 [1.3–9.7], IFN*γ* 5.9 [1.8–9.6]).

### *In vitro* treatment with TNFα, individually and in combination with IFNγ, caused loss of complexes I and IV, which was alleviated by IL-4

**Figure 4:**
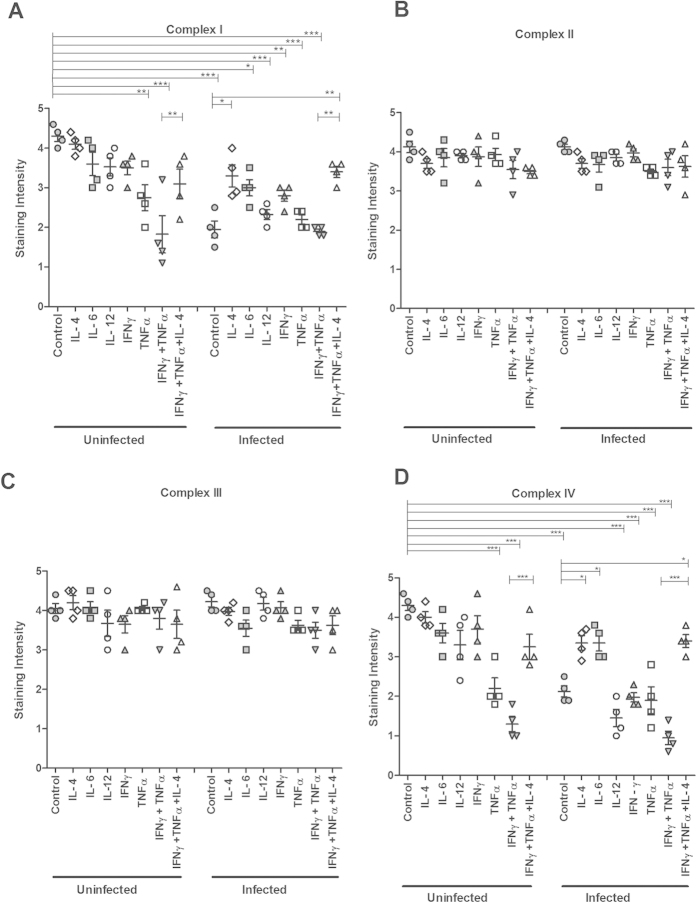
Semi-quantification of mitochondrial respiratory enzyme complexes of an *in vitro* intestinal model treated with cytokines and *C. rodentium* infection. Immunohistochemical staining using antibodies for (**A**) MTND6 (complex I) (**B**) SDHA (complex-II) (**C**) CYC1 (complex-III) (**D**) CCO-VIc (complex-IV). Values are mean ± S.E.M. Statistics: ANOVA with Student Newman-Keuls Multiple Comparison post hoc test: *P < 0.05, **P < 0.01, ***P < 0.001.

We recently developed a semi-wet interface culture method that in combination with mechanical and chemical stimulation creates an *in vitro* mucosal surface with polarised cells, functional tight junctions, a three-dimensional architecture and a mucus layer^18^. We treated this surface with cytokines for 96 h to mimic the extended period of elevated cytokine stimulus that occurred during the infection (Table 1). Immunohistochemical staining indicated that the levels of complexes I-IV remained largely unaffected by IL-4, IL-6, IL-12 and IFN*γ* (Fig. 4). Furthermore, no difference in intensity of complex II and complex III staining was observed in any of the other cytokine treatments performed (Fig. 4B,C). In contrast, TNF*α* caused a marked loss of complex-I and IV staining intensity (*P* < 0.01 and *P* < 0.001, Fig. 4A,D). Combining treatments of TNF*α* and IFN*γ*, in analogy with the *in vivo* cytokine expression during day 14 and 19 post infection, further decreased the intensity of the complex-I and IV staining, but this loss was alleviated by simultaneous treatment with IL-4 (*P* < 0.01 vs *P* < 0.001, Fig. 4A,D).

### *In vitro* reduction of the protein levels of complexes I and IV caused by *C. rodentium* or ETEC infection was alleviated by IL-4

**Figure 5:**
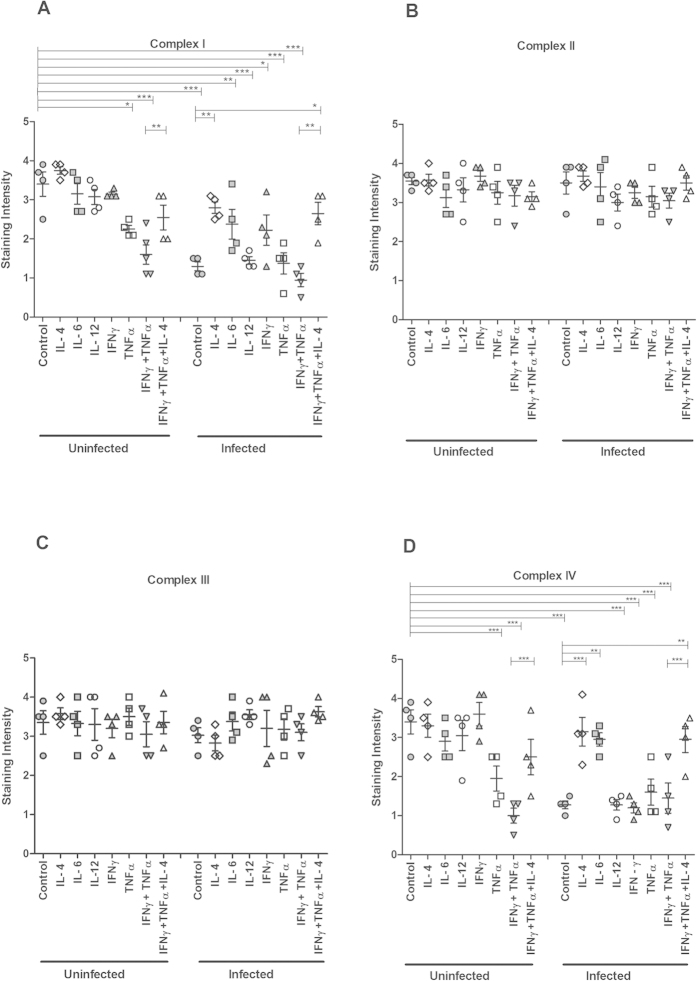
Semi-quantification of mitochondrial respiratory enzyme complexes of an *in vitro* intestinal model treated with cytokines and *E. coli* infection. Immunohistochemical staining using antibodies for (**A**) MTND6 (complex I) (**B**) SDHA (complex-II) (**C**) CYC1 (complex-III) (**D**) CCO-VIc (complex-IV). Values are mean ± S.E.M. Statistics: ANOVA with Student Newman-Keuls Multiple Comparison post hoc test: *P < 0.05, **P < 0.01, ***P < 0.001.

The transepithelial resistance of the *in vitro* mucosal surface remained unaffected after infection with *C. rodentium* (pre infection: 226 ± 22 Ω, 24 h post infection: 233 ± 23 Ω), indicating that the membranes were intact, although some bacteria had translocated across the membrane and were found in the basolateral compartment. Infection caused loss of staining for complex I and IV (*P* < 0.001 vs *P* < 0.001) but not for complex II and III (Fig. 4). IL-4 treatment reversed the infection-induced loss of staining for complex I and IV (*P* < 0.05, Fig. 4A,D), and IL-6 provided protection against loss of complex IV (*P* < 0.05, Fig. 4D). To investigate if other intestinal pathogens could have similar effects, we infected the *in vitro* mucosal surface with enterotoxigenic *E. coli* (ETEC), a human pathogen that lacks the type III secretion system and do not cause A/E lesions. ETEC infection decreased the transepithelial resistance of the *in vitro* mucosal membranes (pre infection: 210 ± 51 Ω, 24 h post infection: 134 ± 12 Ω), but still very similar results were obtained when ETEC was used as the infecting agent instead (Fig. 5). Together, these results indicate that infection, IFN*γ* and TNF*α* have negative effects on mitochondrial respiration, which is alleviated by IL-4, while IL-6 afforded some protection, but only against loss of complex-IV. For further mitochondrial functional studies we therefore focused on *C. rodentium* infection, IFN*γ*, TNF*α* and IL-4.

### Effects on enzymatic activity of the mitochondrial respiratory complexes I and IV caused by *C. rodentium* infection, TNFα and IFNγ, was alleviated by IL-4

**Figure 6:**
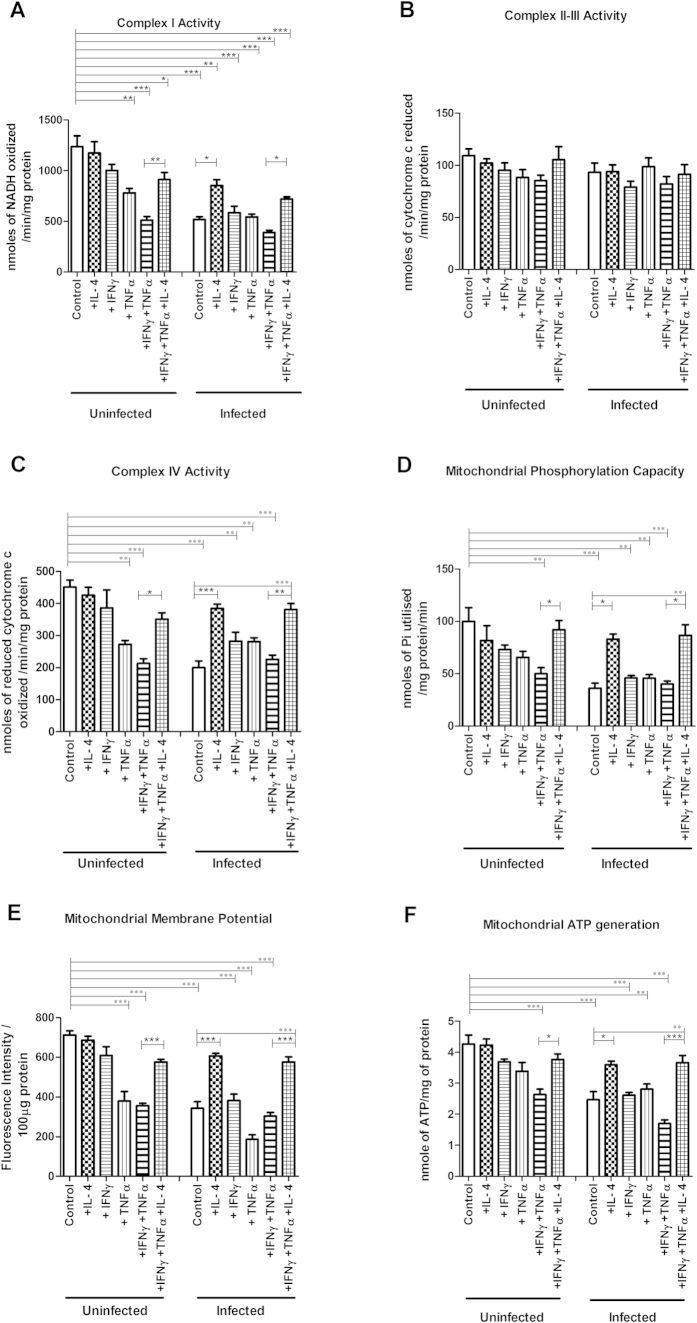
Mitochondrial function of an *in vitro* mucosal intestinal model treated with cytokines and *C. rodentium* infection. (**A**) complex-I activity (**B**) complex-II-III activity (**C**) complex-IV activity (**D**) mitochondrial phosphorylation capacity (**E**) mitochondrial membrane potential (**F**) mitochondrial ATP generation. Values are mean ± S.E.M. Statistics: ANOVA Student Newman-Keuls Multiple Comparison post hoc test: *P < 0.05, **P < 0.01, ***P < 0.001.

In line with the immunohistochemistry results, complex I-IV activities remained unaffected by IL-4 and IFN*γ*, and complex II and III activities were also not affected by TNF*α* and IFN*γ* treatments (Fig. 6). In contrast, TNFα reduced complex I and IV activity (−36% and −39%, *P* < 0.01, Fig. 6A,C). Combining treatments of TNF*α* and IFN*γ*, in analogy with the *in vivo* cytokine expression during day 14 and 19 post infection, further decreased complex I and IV activity (−58% and −52%, *P* < 0.001, Fig. 6A,C). IL-4 reversed the combined inhibitory impact of TNF*α* and IFN*γ* on complex I and IV activity (−58% to −28%, *P* < 0.01, Fig. 6A and −52% to −22%, *P* < 0.05, Fig. 6C).

Infection with *C. rodentium* alone led to decreases in complex I and IV activities that were counteracted by IL-4 (−58% to −13%, *P* < 0.05, and −55% to −14%, *P* < 0.001, Fig. 6A,C). Infection did not exacerbate the reduction of complex I and IV enzymatic activity caused by TNF*α* alone or in combination with IFN*γ*, and IL-4 provided similar protection against the detrimental effects of this combination in the presence of infection (from −68% to −41%, *P* < 0.05, and −49% to −15%, *P* < 0.01, Fig. 6A,C). Infection did, however, decrease the enzymatic activity of both of these complexes in cells treated with IFN*γ* (*P* < 0.01, Fig. 6A and *P* < 0.05, Fig. 6C). Complex II-III activity was not affected by infection with or without cytokine treatment.

### *In vitro*, IL-4 counteracted the decreases in mitochondrial phosphorylation capacity, transmembrane potential and ATP generation caused by *C. rodentium* infection, TNFα and IFNγ

Reflecting the loss of complex I and IV activity, the mitochondrial phosphorylation capacity was hampered by TNF*α* and IFN*γ* both in the absence (−50%, *P* < 0.01) and presence (−60%, *P* < 0.001) of *C. rodentium* infection (Fig. 6D). IL-4 alleviated this impairment (*P* < 0.05) to a degree that it was not statistically different from non-treated mucosal membranes (Fig. 6D). Infection *per se*, and also in combination with TNF*α* and IFN*γ*, caused a reduction in mitochondrial phosphorylation capacity (Fig. 6D). IL-4 alleviated the impairment of the mitochondrial phosphorylation caused by the cytokines and infection, together or alone, to a degree similar to non-treated mucosal membranes (Fig. 6D). The impact of cytokines and *C. rodentium* infection on mitochondrial membrane potential (Fig. 6E) and ATP generation (Fig. 6F) followed a very similar pattern. Thus, IL-4 alleviated the detrimental effect of TNF*α* and IFN*γ* on ATP generation in both uninfected (from −38% to −11%, *P* < 0.05) and infected (from −60% to −14%, *P* < 0.001, Fig. 6F) conditions, and negated the direct impact of *C. rodentium* infection, reviving the mitochondrial ATP generation from 58% to 85% (*P* < 0.05).

### *In vivo*, the levels of complex I and IV are more affected by the cytokine environment than by pathogen density

To elucidate the role of the cytokine environment versus the direct actions of *C. rodentium in vivo*, we studied IFN*γ*−/− mice, as IFN*γ* increased early in infection, concomitantly with the mitochondrial dysfunction (in contrast to TNF*α*). IFN*γ*−/− mice had a similar *C. rodentium* burden to that of the WT mice at day 10 post infection (mean ± SEM: Log 6,5 ± 0,2 CFU/g feces for IFN*γ*−/− and Log 6,6 ± 0,3 CFU/g feces for WT, n = 7) while at day 14 post infection the density was slightly higher in the IFN*γ*−/− mice (*P* < 0.05, Log 4,8 ± 0,3 CFU/g feces) than in the WT (mean Log 3,4 ± 0,3 CFU/g feces) mice. The cytokine environment during the course of infection was different in IFN*γ*−/− compared to WT mice, mainly with regards to that IL-4 and IL-6 were upregulated already by day 10 post infection (3-fold vs 20-fold, Table 1).

**Figure 7:**
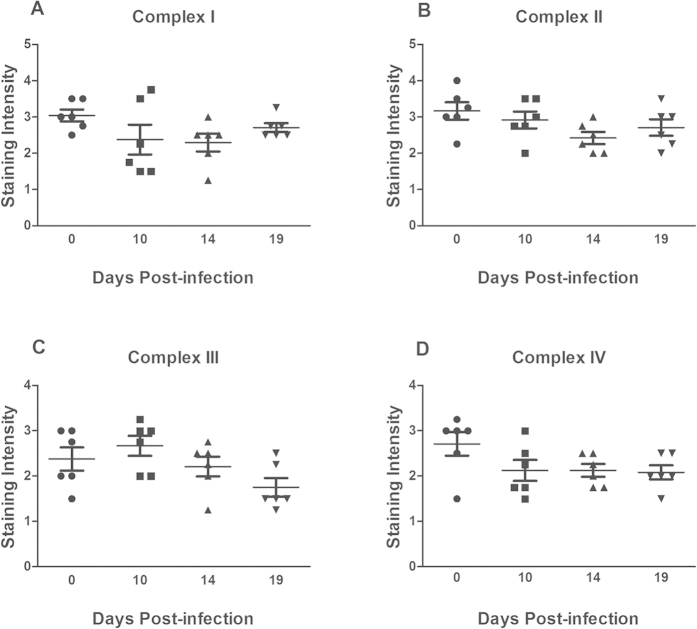
Semi-quantitative analysis of the mitochondrial respiratory enzyme complexes in the distal colon of IFNγ−/− mice after *C. rodentium* infection. Immunohistochemical staining using antibodies for (**A**) MTND6 (complex I) (**B**) SDHA (complex-II) (**C**) CYC1 (complex-III) (**D**) CCO-VIc (complex-IV). Statistics: ANOVA Student Newman-Keuls Multiple Comparison post hoc test: *P < 0.05, **P < 0.01, ***P < 0.001. vs. control.

All four complexes (complex I–IV) were present relatively uniformly in the majority of the epithelial cells in the colon of IFN*γ*−/− mice, with a similar tissue location and staining intensity as in the WT mice (compare the non-infected controls in Figs 2 and 7). In contrast to the loss of staining intensity of subunits of complex I and IV found in colons from WT mice after infection (Fig. 2), there was no statistically significant loss of any of the four complexes in IFN*γ*−/− mice (Fig. 7A–D). Thus, it appears that *in vivo*, the cytokine environment is a more important determinant of mitochondrial complex levels than the direct actions of the pathogen. This is further supported by the observation that in the WT mice, the mitochondrial respiratory chain remained impaired even at day 19 post infection, when the pathogen burden had subsided, but the expression of TNF*α* and IFN*γ* remained elevated (Figs 2 and 3). Although the caspase-3 levels in the colonic tissue from IFN*γ*−/− mice were slightly elevated at day 14 post infection (p < 0.01), the magnitude of the increase was less than in the wt mice (p < 0.01, Fig. 1I), and none of the other time points showed an increase, whereas all time points in the WT animals had statistically significant increases.

### *In vivo*, the cytokine environment, and not the *C. rodentium* density, determine the level of 3-Nitrotyrosine

**Figure 8:**
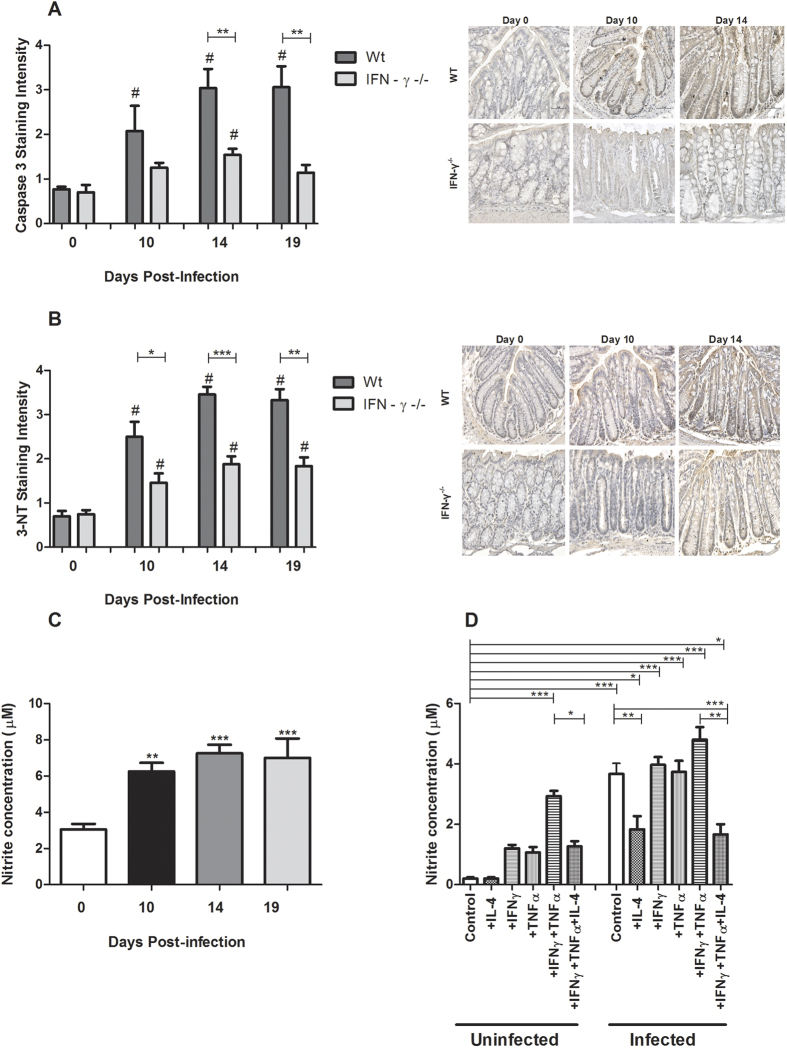
Caspase-3, 3-NT staining scores and nitrite measurements after *C. rodentium* infection. (**A**) The caspase-3 staining scores based on immunohistochemical staining and corresponding representative photos of the caspase-3 tissue localization in the distal colon of WT and IFNγ−/− mice. Values are mean ± S.E.M. Statistics: ANOVA with Newman-Keuls Multiple Comparison post hoc test, ^#^*P* < 0.05 vs. corresponding non-infected genotype control, unpaired t test **P < 0.01 compared to WT of same infection period (n = 4–9 mice/group). Scale bar 50 μm, magnification × 400. (**B**) Immunohistochemical staining scores and photos representing the tissue localization of the 3-NT residues in the murine distal colons of WT and IFNγ−/− mice during infection. Scale bar 50 μm, magnification × 400. Statistics: ANOVA with Student Newman-Keuls Multiple Comparison post hoc test: ^#^*P* < 0.05 vs. corresponding genotype control, unpaired t test: *P < 0.05, **P < 0.01, ***P < 0.001 vs. WT at the same time point of infection, n = 4–9 mice/group. (**C**) Nitrite concentration in the murine distal colon after *C. rodentium* infection. Statistics: ANOVA with Student Newman-Keuls Multiple Comparison post hoc test: *P < 0.05, **P < 0.01, ***P < 0.001. vs. control. (**D**) Nitrite concentration in the *in vitro* mucosal intestinal model treated with cytokines and *C*. *rodentium* infection. Statistics: ANOVA with Student Newman-Keuls Multiple Comparison post hoc test: *P < 0.05, **P < 0.01, ***P < 0.001.

Infection with *C. rodentium* resulted in an increase in immunostaining intensity for 3-Nitrotyrosine (3-NT), a marker for oxidative damage, in both infected WT and IFN*γ*−/− mice at all time points post-infection (p < 0.05–0.0001, Fig. 8B). However, the levels of 3-NT were higher in WT mice compared to IFN*γ*−/− mice, with the day 14 and 19 timepoints in the WT having intensity scores twice as high as the IFN*γ*−/− mice (p < 0.05, Fig. 8B). Since IFN*γ*−/− mice had a similar pathogen burden, but a cytokine environment without IFN*γ* but higher in IL-4 and IL-6, this suggests that the cytokine environment, and not the pathogen burden, is the main cause of the NO generation *in vivo*.

### *In vivo* and *in vitro*, NO generation increased during *C. rodentium* infection and increased levels of TNFα and IFNγ, which was counteracted by IL-4

In line with the above results, infection with *C. rodentium* resulted in increased generation of NO_2_^−^, another index for oxidative damage, in WT mice in all time points post-infection (P < 0.001, Fig. 8C). In the non-infected *in vitro* mucosal membranes, the combined action of IFNγ and TNFα led to the highest generation of NO_2_^−^ (p < 0.001, Fig. 8D), and this was alleviated by IL-4 treatment (p < 0.05, Fig. 8D). *In vitro* infection further increased the generation of NO_2_^−^, and IL-4 alleviated both the NO_2_^−^ generation-induced by the bacteria alone (p < 0.01, Fig. 8D) and by the combined actions of infection, IFNγ and TNFα (p < 0.01, Fig. 8D). Although the higher level of the NO_2_^−^ generation in the infected *in vitro* membranes may at first glance appear to contradict the *in vivo* results demonstrating that the cytokine environment is a more important determinant of NO-levels than the pathogen density *in vivo*, these results are not surprising since *in vitro, C. rodentium* multiplies unhindered, and the bacterial density after 24 h of co-culture is higher than *in vivo*.

### NO generation inversely correlated with mitochondrial function

In *C. rodentium* infected mice, the level of NO_2_^−^ inversely correlated with complex I and complex-IV activities (Pearson product-moment correlation coefficient r^2^ = −0.823, p < 0.01 and r^2^ = −0.714, p < 0.01, respectively), mitochondrial phosphorylation (r^2^ = −0.846, p < 0.01), mitochondrial membrane potential (r^2^ = −0.735, p < 0.01) and ATP generation (r^2^ = −0.669, p < 0.01, compare Figs 8C and 3). Similarly, after *in vitro* cytokine treatment and *C. rodentium* infection, the level of NO_2_^−^ inversely correlated with complex I and complex-IV activities (r^2^ = −0.851, p < 0.01 and r^2^ = −0.733, p < 0.01 respectively), mitochondrial phosphorylation (r^2^ = −0.744, p < 0.01), mitochondrial membrane potential (r^2^ = −0.782, p < 0.01) and ATP generation (r^2^ = −0.829, p < 0.01, Fig. 8D compared with Fig. 6).

### *In vivo* and *in vitro*, mitoquinone (MitoQ) alleviated the damaging impact on mitochondrial function during *C. rodentium* infection

**Figure 9:**
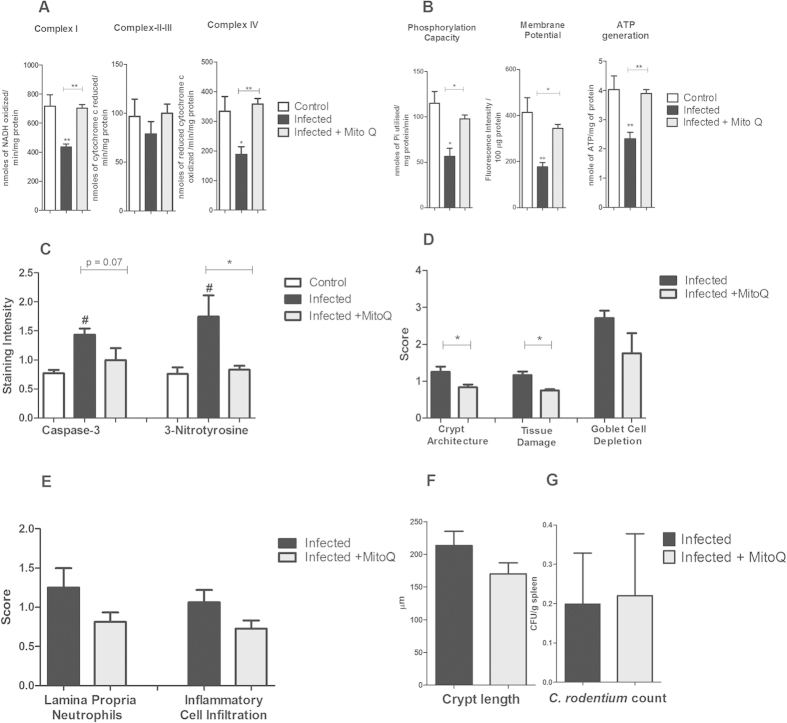
Effects of MitoQ on mitochondrial functions, caspase-3, NO, colitis and *C. rodentium* translocation. (**A**) complex I, II–III and IV activities and (**B**) mitochondrial phosphorylation capacity, membrane potential and mitochondrial ATP generation in infected and MitoQ treated mice. Values are mean ± S.E.M. Statistics: ANOVA with Student Newman-Keuls Multiple Comparison post hoc test: *P < 0.05, **P < 0.01, ***P < 0.001 (n = 4/group). (**C**) caspase-3 and 3-Nitrotyrosine immunostaining in infected and MitoQ treated mice. Values are mean ± S.E.M. Statistics: ANOVA with Student Newman-Keuls Multiple Comparison post hoc test: *P < 0.05 vs non-infected control, ^#^*P* < 0.05 vs. infected control. (**D**) colonic scores of crypt architecture, tissue damage and goblet cell depletion, (**E**) Neutrophils in lamina propria and inflammatory cell infiltration, (**F**) crypt length and (**G**) *C. rodentium* counts in spleen of infected and MitoQ treated mice. Values are mean ± S.E.M. (n = 4 mice/group). Statistics: paired t test, *P < 0.05 compared to infected mice.

**Figure 10:**
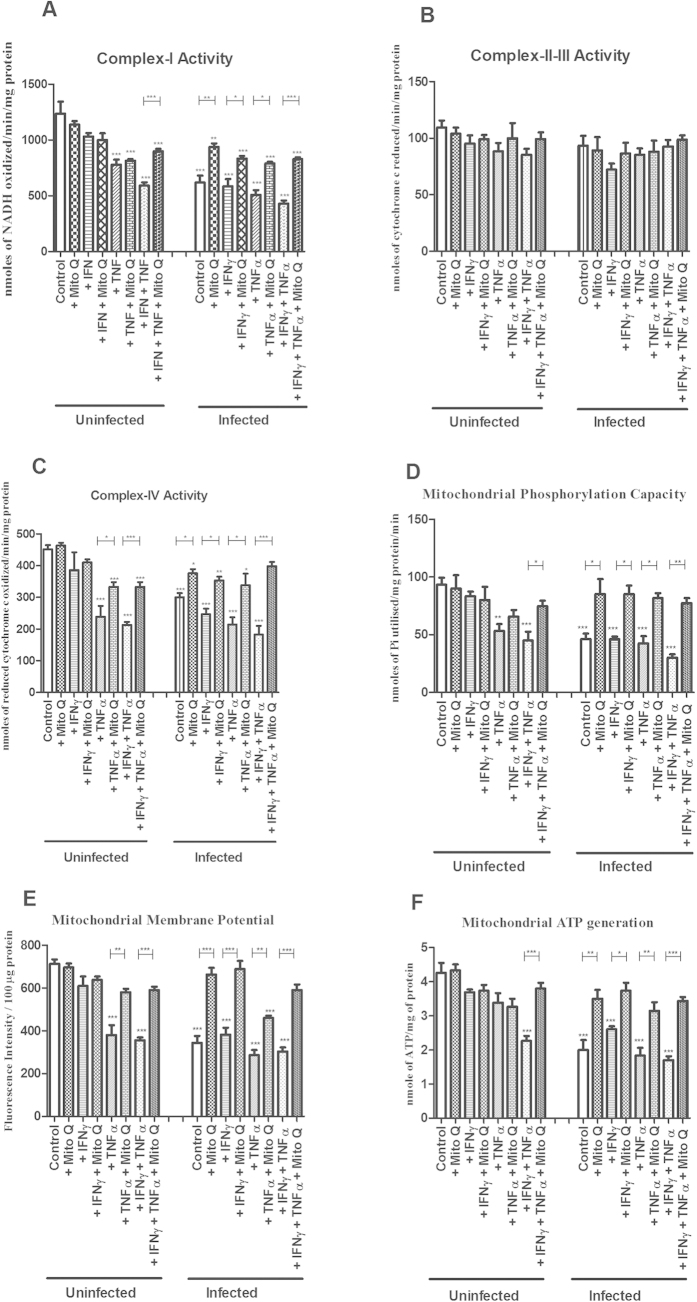
Effects of MitoQ on mitochondrial functions in the *in vitro* mucosal intestinal model treated cytokines with/without *C. rodentium* infection. (**A**) complex-I activity, (**B**) complex-II–III activity, (**C**) complex-IV activity, (**D**) mitochondrial phosphorylation capacity, (**E**) mitochondrial membrane potential, and (**F**) mitochondrial ATP generation. Values are mean ± S.E.M. Statistics: ANOVA with Student Newman-Keuls Multiple Comparison post hoc test: *P < 0.05, **P < 0.01, ***P < 0.001.

MitoQ is an antioxidant that accumulates within mitochondria, and that has been used in clinical trials in humans^25^. Treating mice with established infection with MitoQ (from day 5 to 14) restored the complex-I and complex-IV activities, mitochondrial phosphorylation, membrane potential and ATP generation (Fig. 9A,B). Furthermore, MitoQ treatment reverted the infection-induced 3-nitrotyrosine staining (p < 0.05, Fig. 9C). In line with these results, MitoQ also alleviated the damaging influence of infection, TNFα and IFNγ on these parameters *in vitro* (Fig. 10A–F).

Although treatment with MitoQ restored all of the mitochondrial parameters, not all features of the disease were improved. Crypt architecture and tissue damage improved (*P* < 0.05 for both, Fig. 9D), the caspase-3 levels decreased to an extent where it was not statistically different from uninfected controls, and the goblet cell depletion trended towards a decrease (p = 0.077, Fig. 9C–F). However, the number of *C. rodentium* in feces and spleen was similar to that of infected mice without MitoQ treatment (Fig. 9G).

## Discussion

In the present study, we demonstrate for the first time that infection reduces mitochondrial complex I and IV protein levels and enzymatic activity and also phosphorylation capacity, transmembrane potential and ATP generation throughout infection and clearance of the pathogen. While previous studies have shown that bacteria can affect mitochondrial function, the endpoints examined were a loss of mitochondrial membrane potential or involvement in apoptosis induction; only one study went further and looked at complex II expression after infection^13^. Using an *in vivo*-like *in vitro* mucosal surface, we identified that infection *per se*, as well as TNF*α*, individually and more severely in combination with IFN*γ*, caused the same effects as seen *in vivo*. Co-treatment with IL-4, however, reversed these responses, and IL-6 also protected against loss of complex IV. The negative effects on mitochondria were caused largely by NO generation, and were reversed by mitochondrial antioxidant treatment. IFN*γ*−/− mice, which had a similar pathogen burden as infected WT mice, but a colonic cytokine environment with higher levels of IL-4 and IL-6, displayed no loss of any of the four complexes, demonstrating that the effects on mitochondria found *in vivo* were largely cytokine driven. Thus, as the concentration of IFN*γ* and TNF*α* increase during the latter time points of infection and clearance in WT mice, the concomitant increase of IL-4 and IL-6 appears to protect the mitochondrial functions of the colonic epithelium.

Of the four multimeric complexes involved in mitochondrial respiratory phosphorylation, inhibition in both levels and activities of complex I and complex IV were observed at all post-infection time points. Although the extraction process may stress the mitochondria and therefore possibly alter their function, we here provide two lines of evidence that support the results, whereof the first does not involve extraction: *in situ* quantification using immunohistochemistry (showing decreased levels of complex I and IV) and functional assays (demonstrating decreases in the activity of complexes I and IV, phosphorylation capacity, transmembrane potential and ATP generation). No inhibition in activity of complex II–III was noticed at any time-points. However, we did find a reduction in immunohistochemical staining intensity for complex II, which was in agreement with the earlier study^13^. This decrease in quantity while simultaneously retaining normal specific activity of succinate cytochrome c reductase (indicator for complex II–III activity) post-infection is puzzling. A reason for this discrepancy may be that the method adapted for measuring the activity of succinate cytochrome c reductase measures the enzymatic activity of both complex II and III, thus it is possible that normal complex III activity can mask the inhibition of complex II activity, although the method here is widely used and demonstrated to be suitable to detect complex II deficiency^26^. Another reason could be that a decrease in the level of enzyme can be compensated for by an increase in activity^26^.

Targeting of the host cell mitochondria appears to be a common strategy among many clinically important pathogens. Bacteria like *Neisseria gonorrheae*^27^, *Neisseria meningitides*^28^, *Helicobacter pylori*^29^ and *Salmonella enterica* serovar Typhimurium^30^, all target host cell mitochondria by translocating proteins that trigger cell death, mainly through apoptosis. Similarly, one report indicated involvement of *C*. *rodentium* effector proteins in causing cell death through affecting mitochondrial membrane potential and succinate dehydrogenase levels^17^. Cell death at the base of the colonic crypts, where the presence of *C. rodentium* is unlikely, has also been reported^5^, and our caspase-3 staining results further confirmed a similar incidence of cell death both on the luminal surface and in the crypts during infection. As cytokines have the capability to regulate epithelial cell function irrespective of the position of the cells in the crypt, we investigated the impact of cytokines that changed expression after infection, on mitochondrial function. Treatment with TNF*α in vitro* decreased mitochondrial functional parameters and the levels and activity of the complex I and IV enzymes, and when combined with IFN*γ* had an even greater effect. Even though IFN*γ* is known to be an immunoregulatory cytokine promoting immune responses at the initiation of several bacterial infections^31^,^32^,^33^,^34^, the impact of IFN*γ* on mitochondrial function during infections has not been described previously. TNF*α*, alone as well as together with IFN*γ*, has previously been shown to affect mitochondrial function in non-intestinal tissues under different experimental conditions^35^,^36^,^37^,^38^,^39^.

The protective effects of IL-4 on complex I and IV activity and levels that we observed *in vitro* is consistent with its previously observed ability to abrogate cell death by maintaining mitochondrial membrane potential and anti-oxidant status in other systems, such as B-cells^40^. To conclusively prove that IL-4 and IL-6 provide the same function during infection *in vivo* is more complicated, as changing their levels alter a whole range of infection related parameters. Indeed, IL-6 deficient mice have been shown to have high mortality and 100-fold higher pathogen burdens compared to WT mice during *C. rodentium* infection^41^. However, our results from IFN*γ*−/− mice supports our proposal that IL-4 and IL-6 protects the mitochondria during infection as these mice, which had higher levels of IL-4 and IL-6 but a similar pathogen burden to WT mice, had no reduction of the levels of any of the four complexes during *C*. *rodentium* infection, and greatly reduced NO generation. As loss of mitochondrial ATP generation is the consequence of dysfunction of the mitochondrial respiratory chain, these results are in line with our observation in WT animals that there are high levels of caspase-3 staining, as well as high numbers of dead and sloughed off cells during clearance of infection even at day 19 post infection, when the pathogen burden is almost entirely absent^19^, but the expression of IFN*γ* and TNF*α* remains high. In WT mice, when the concentrations of IFN*γ* and TNF*α* increase during clearance of infection, the concurrent induction of IL-4 and IL-6 thus appears to protect the mitochondrial function of the colonic epithelium from further damage.

Our results suggest that NO generation caused by infection-induced TNF*α* and IFN*γ* was the main cause of mitochondrial dysfunction. The dysfunction was reversed by IL-4 treatment, indicating that IL-4 has a role in regulating NO production. That the NO pathway plays a substantial role in mitochondrial dysfunction is further verified by the protective effect of the antioxidant MitoQ, which is a scavenger for the peroxynitrite (ONOO^−^) that is generated when NO reacts with superoxide (O_2_^−^)^42^. Furthermore, a previous study has shown that *C. rodentium* infection results in up-regulation of iNOS in the colonic epithelium *in vivo*, and that iNOS^−/−^ mice are protected from *C. rodentium* induced inflammation, including attenuated levels of TNFα and IFNγ^43^. Oxidative insult to the respiratory chain complexes can amplify and promote further oxidative damage^44^, and indeed mitochondrial complexes I and IV, which were most effected in our study, are encoded in the mitochondrial genome^45^ and thereby susceptible to mutations in mitochondrial DNA.

In spite of less epithelial damage after MitoQ treatment and similar levels of *C. rodentium* in colon, the number of *C. rodentium* in the spleen did not improve. However, the number of CFU that are found in the internal organs during this infection is rather low, and possibly the ones that enter do so through mechanisms other than direct translocation across a damaged epithelial membrane. A similar mitochondria targeting antioxidant (MitoTEMPO) was recently shown to inhibit superoxide induced *E. coli* translocation over mucosal membranes *in vitro*^46^. To elucidate if antioxidants with protective effects towards mitochondria could have a role in treating infections, an *in vivo* model where bacterial translocation over the epithelium plays a more prominent role in progression of disease needs to be utilised.

In conclusion, infection with this A/E pathogen induces mitochondrial dysfunction, which is largely caused by IFN*γ* and TNF*α* synergistically compromising complex I and IV levels and activity, via NO generation. *In vitro*, the pathogen *per se* also induces a similar effect, but *in vivo*, the cytokine environment appears to be the dominating factor governing mitochondrial enzyme levels. The presence of both IL-4 and IL-6 during the clearance phase of infection, when TNF*α* and IFN*γ* levels are exceedingly high, protects the colonic epithelial surface against more detrimental damage.

## Methods

### Animals

6–8 weeks old, specific-pathogen-free, male C57BL/6 mice purchased from Taconic (England) or Charles River (Germany). IFN*γ*-deficient mice on a C57BL/6 background were bred and housed in ventilated cages under pathogen-free conditions at the Laboratory for Experimental Biomedicine (EBM), Sahlgrenska Academy, Gothenburg. Mice were fed *ad libitum* and monitored daily.

### Ethics statement

All experimental procedures were approved, and performed in accordance with, the guidelines laid by the Göteborgs Djurförsöksetiska Nämnd (Ethic No. 261/09) based on regulations from Djurskyddsförordningen DFS 2004:4.

### Culture of the *in vitro* colonic mucosal model

For propagation, the human intestinal cell line HT29 MTX-E12 was cultured (at 37 °C, 5% CO_2_ −95% air) in RPMI containing 10% (v/v) FCS, 1% 5000 U (v/v) penicillin-streptomycin (Lonza). To form the *in vitro* colonic mucosal surface^18^, 7.5 × 10^4^ cells in 200 *μ*l of RPMI containing 10% (v/v) FCS and penicillin-streptomycin were added to the apical side of Snapwell membranes (0.4 mm pores) with 12 mm diameter (Corning). When cells became confluent (4–6 days later) they were subjected to semi-wet interface culture with continuous rocking, with 2 ml media in the basolateral compartment and 50 *μ*l of media in the apical compartment for 28 days. Basolateral media was refreshed every two days and for the first 6 days it was supplemented with 10 mM *N*-[(3,5-Difluorophenyl)acetyl]-L-alanyl-2-phenyl]glycine-1,1-dimethylethyl ester (DAPT, Sigma-Aldrich).

### Infection and treatments

Mice: C. *rodentium* strain ICC169 was grown on MacConkey agar (Oxoid) for 20 h at 37 °C. Male C57BL/6 wild type and IFN*γ*−/− mice were infected with 100 *μ*l of bacterial suspension (5 × 10^9^ colony forming units (CFU) in Luria-Bertani (LB) broth) by oral gavage. Infection experiments were performed twice for each time point. MitoQ treatment: infected mice were administered MitoQ orally through drinking water (500 μM) from day 5 to 14 of infection period. This dose of MitoQ was chosen as it was not toxic in an earlier study^47^. Mice were anaesthetised with isoflurane and killed by cervical dislocation at day 10, 14 and 19 post infection. The last 2.5 cm of colon, beginning at the anal verge, was collected. For the first set of experiments for all time points, the most distal 1 cm were harvested into fresh Carnoy’s fixative (60% dry methanol, 30% chloroform, 10% glacial acetic acid); the next distal 1 cm colonic specimens stored in RNAlater (Ambion) for RNA isolation. For the second set of experiments, the most distal 1.5 cm colonic specimens were harvested into Carnoy’s methanol fixative and the next distal 1 cm colonic specimens in ice cold imidazole buffer (50 mM, pH 7.4) for mitochondrial isolation.

*In vitro* cytokine treatments and infection: *In vitro* mucosal surfaces (described above) were treated with cytokines for 96 h, starting on day 28 post confluency. The 96 h duration treatment was to mimic physiological conditions during infection, as colon epithelial cells were exposed to elevated cytokine levels for days (table 1). The cultures were exposed individually and in combination with IFN*γ* (10 ng/ml), TNF*α* (10 ng/ml), IL-4 (1.5 ng/ml), IL-6 (15 ng/ml) and IL-12 (20 ng/ml); these concentrations were based on previous work^41^,^48^,^49^,^50^,^51^,^52^,^53^. For MitoQ treatment, 50 nM was added to the basolateral side for 96 h, the dose was based on previous work^47^. Antibiotic-free RPMI containing 10% FBS, cytokines and/or MitoQ was changed every 24 h^54^,^55^,^56^. For infection, 10 *μ*l of *C. rodentium* and enterotoxigenic *E. coli* (ETEC, strain E2265) suspensions with a respective OD of 2.0 and 0.1 at 410 nm (CFU: 10^7^ and 5 × 10^5^ CFU, corresponding to a multiplicity of infection of 10:1 and 0.5:1 ) in sterile PBS was added to the apical side of the membrane 24 h prior to the experimental end point. Although the epithelial surface is exposed to bacteria for days *in vivo*, this was not technically possible *in vitro*, due to overgrowth of the bacteria. To monitor the effects of infection on the membranes, Trans Epithelial Electrical Resistance (TEER) was measured using an EVOM^2^ meter and STX2 probe (World Precision Instruments, Sarasota, Florida, USA).

Histology-For colitis analysis in infected mice, 5 *μ*m sections of Carnoy’s fixed tissue were stained with haematoxylin/eosin, coded to blind the analysis, and the entire section was systematically scored for: aberrant crypt architecture (0–3), tissue damage (0–3), increased crypt length (0–3), goblet cell depletion (0–3, confirmed by PAS/Alcian blue stain^18^), lamina propria neutrophil counts (0–3), crypt abscesses (0–3) and inflammatory cell infiltration (0–3).

### RT-PCR for cytokines

For the quantitative RT-PCR cytokine array, total RNA was extracted from distal colon using the RNeasy mini kit (QIAGEN), and cDNA prepared using the QuantiTect Reverse Transcription kit (QIAGEN). mRNA from two sets of two mice in each group were pooled for the time points of day 0 and day 10 (i.e. data representative of four mice in each group), while time points day 14 and 19 contained mRNA pooled from three mice in each group. RT-PCR on pooled samples was carried out in duplicate using RT^2^ Profiler™ PCR Array (PAMM-034Z) plates containing 84 mouse inflammatory cytokine, chemokine and receptor genes (QIAGEN). The arrays were run on an ABI 7500 real-time PCR system (Applied Biosystems). Intra-plate controls were included and data were normalised by the RT2 Profiler PCR Array data analysis software (QIAGEN) using the most suitable housekeeping gene chosen from five housekeeping genes [*Gusb* (Glucuronidase, beta), *Hprt1* (Hypoxanthine guanine phosphoribosyl transferase1), *Hsp90ab1* (Heat shock protein 90 kDA alpha (cytosolic), class B member 2), *Gapdh* (Glyceraldehyde-3-phosphate dehydrogenase), *Actb* (Actin, beta cytoplasmic)] present in the plates, with a threshold of variance of 0.2 cycles. Fold changes against control mice were calculated using the same software. Fold changes ≥2.5 were accepted as up- or downregulation. For the individual RT-PCRs, total RNA was extracted from three uninfected WT control mice and three mice from day 19 post infection using TRizol (Life Technologies, Carlsbad, CA, USA). RNA purity was assessed through UV spectroscopy (NanoDrop; Thermo Scientific, MA, USA). Total RNA (5 μg) was treated with DNase at 37 °C for 30 min, addition of 5 mM EDTA and heat inactivation of DNase at 75 °C for 10 min followed by cDNA synthesis. A final concentration of 5 mM MgCl_2_ was added to RNA samples, which were later used for cDNA synthesis by adding oligo-dT primers and Superscript III (Life Technologies, Carlsbad, CA, USA) at 50 °C for 2 h. The cDNA was used in a RT-PCR using Evagreen SSO-Fast (Bio-Rad laboratories, Hercules, CA, USA) and IL-4 (Fwd: GGCTTTTCGATGCCTGGATT, Rev: TTTGCATGATGCTCTTTAGGCTTT), TNFα^57^ and IFNγ primers^57^. The expression of *hprt1* (QIAGEN) and *eif2* (Eukaryotic initiation factor 2) (designed using Primer3 program http://frodo.wi.mit.edu/primer3/ Fwd:GCTTCCCTGTTCACCTCTGA, Rev: CACATGGGCGATGACTGAC) reference genes were used for normalizing the qPCR data. Samples were amplified in triplicate with a negative control without reverse transcriptase to confirm the lack of contaminating genomic DNA. Data acquisition and analysis were performed using CFX manager 3.1 software (Bio-Rad Laboratories Inc., Hercules, CA, USA).

### Immunohistochemistry

Antigen retrieval was performed using Dako target retrieval solution, pH 9.0 (S2367, Dako) 30 min (murine samples) or 15 min (*in vitro* mucosal surface) at 95 °C then cooled in room temperature for 40 min. Endogenous peroxidase activity was blocked using 3% hydrogen peroxide in PBS, 15 min and non-specific antibody binding prevented by blocking in Dako protein block serum-free reagent (X0909, Dako) for 30 min, room temperature prior to incubation with primary antibodies. Rabbit polyclonal primary antibodies for subunits of the mitochondrial respiratory enzyme complexes were selected for complex I (anti-NADH dehydrogenase subunit 6, MTND6, orb6548, Biorbyt), complex-II (anti-Succinate dehydrogenase subunit A, SDHA, ab86932, Abcam), complex-III (anti-Ubiquinol-cytochrome c reductase complex cytochrome c1 subunit, CYC1, NBP1-86872, Novus Biologicals), complex-IV (anti-Cytochrome c oxidase subunit VIc ,CCO-VIc, ab150422, Abcam), Caspase-3 (activated form, ab4051, Abcam) and 3-Nitrotyrosine (AB5411, Millipore) at optimal dilutions of 1:2000, 1:200, 1:2000, 1:2000, 1:500 and 1:500 respectively, in antibody diluent (Dako, S0809). Incubation with the complex-II (SDHA) and Caspase-3 primary antibodies was performed overnight at 4 °C; incubation with complex I, III, IV and 3-Nitrotyrosine antibodies were for 3 h at 23 °C. Sections were then washed four times in PBS containing 0.05% Tween 20, then incubated with a horseradish peroxidase (HRP) labelled anti-rabbit polymer for 15 min using a reagent kit from Dianova GmbH (PT03-L). Immunostaining was visualised using 0.05% 3,3′-diaminobenzidine hydrochloride (DAB) chromogen (CD-12, Dako) for 10 min, then counterstained with haematoxylin. Scoring of staining intensity was performed blinded, and scores of (blinded) key samples were verified by a second independent observer. Scores represent the average of 1 cm distal colon, using a scale of 0–5.

### Isolation of the mitochondrial fraction

Murine colon: Specimens collected in ice cold imidazole buffer (50 mM, pH 7.4) were scraped to collect epithelial cells in a petri dish on ice. Epithelial scrapings were suspended in 5 ml of homogenizing buffer A [225 mM mannitol, 75 mM sucrose, 5 mM 4-(2-hydroxyethyl)-1-piperazineethanesulfonic acid (HEPES), 1 mM ethylene glycol tetraacetic acid (EGTA), 1 mg/ml bovine serum albumin (BSA, pH 7.4)] and homogenised with a tissue homogenizer (VWR-VDI12,VWR) using 7–10 strokes. The homogenate was brought to 15 ml with the same buffer and centrifuged at 1000 *g* for 10 min at 4 °C. The supernatant was saved, the pellet was resuspended in homogenizing buffer A and centrifuged again at 1000 *g* for 10 min at 4 °C. Supernatants from these two steps were pooled and centrifuged at 10000 *g* for 10 min at 4 °C. The supernatant was stored at −20 °C for the nitrite assay and the pellet was utilised for mitochondrial isolation. The pellet was resuspended in homogenization buffer A without EGTA and BSA, then centrifuged at 10000 *g* for 10 min at 4 °C. For measurement of mitochondrial respiratory complex activities, mitochondria were resuspended in 50 mM phosphate buffer, pH 7.4; for measurement of membrane potential, mitochondrial ATP and phosphorylation capacity, the final mitochondrial pellet was resuspended in an isotonic buffer (145 mM KCl, 50 mM sucrose, 1 mM EGTA, 1 mM magnesium chloride, 10 mM phosphate buffer, pH 7.4). All aliquots of mitochondrial suspensions were kept frozen at −20 °C and used within a week.

### *In vitro* mucosal surface

The method of Modica-Napolitano *et al*. 1989^58^ with minor modifications was used: Cells from 12 membranes, pooled into 3 replicates (4 membranes per sample) per combination of treatment regimen were harvested in culture medium, pelleted, and washed with homogenization buffer B (250 mM sucrose, 1 mM Tris-HCI, l mM ethylene diaminetetraacetic acid (EDTA, Sigma-Aldrich), 1 mg/ml of BSA, pH 7.4) at 4 °C. The cells were resuspended in 5 ml of ice-cold homogenization buffer B, homogenised with 110 strokes to disrupt at least 95% of the cells^58^, centrifuged at 1000 *g* for 10 min at 4 °C. The supernatant was saved, the pellet resuspended and centrifuged again at 1000 *g* for 10 min at 4 °C. The supernatants from these two steps were pooled and centrifuged at 10000 *g* for 10 min. The supernatant obtained was stored at −20 °C for the nitrite assay and the pellet resuspended in homogenization B buffer containing digitonin (0.02%), then centrifuged again at 10000 *g*, and finally resuspended in an appropriate buffer for further experimentation (same as for mitochondria from colon tissue, above).

### Assessing mitochondrial respiratory enzyme complex activities

The complex activities in the mitochondrial fractions were measured following standard protocols: NADH-ferricyanide reductase (complex-I) activity was measured following the method of Hatefi^59^ using ferricyanide as the electron acceptor in a system containing 0.17 mM NADH, 0.6 mM ferricyanide, and Triton X-100 (0.1% v/v) in 50 mM phosphate buffer, pH 7.4 at 30 °C. The reaction was initiated with addition of the mitochondrial suspension (10 *μ*g protein), and the rate of oxidation of NADH was measured by the decrease in absorbance at 340 nm.

The activity of succinate cytochrome c reductase (complex II–III) was assayed by following the succinate supported reduction of ferricytochrome c to ferrocytochrome c at 550 nm in an assay mixture containing 100 mM phosphate buffer, 2 mM succinate, 1 mM KCN, 0.3 mM EDTA and 1.2 mg/ml cytochrome c (Sigma-Aldrich) in a total volume of 0.4 ml^60^. The reaction was initiated by adding mitochondrial suspension (10 *μ*g protein) to the sample cuvette. The same assay was repeated with (10 *μ*M) antimycin (Sigma-Aldrich) to determine the inhibitor sensitive rate and the results were expressed as nmoles of cytochrome c reduced/min/mg protein.

The activity of cytochrome c oxidase (complex IV) was assayed by measuring the rate of decrease of absorbance at 550 nm at room temperature following the oxidation of reduced cytochrome c (50 *μ*M) in 10 mM phosphate buffer, pH 7.4^61^. Ferricyanide (1 mM) was added to oxidize ferrocytochrome c in the blank cuvette and the reaction initiated in the sample cuvette by the addition of mitochondrial suspension (10 *μ*g). The activity of the enzyme was expressed as nmoles of cytochrome c oxidised/min/mg protein.

### Measurement of mitochondrial phosphorylation capacity

Phosphate utilization was assayed following a method published previously^62^. In a total volume of 250 *μ*l, an aliquot of 25 *μ*l mitochondrial suspension was diluted into a medium containing 125 mM KCl, 75 mM sucrose, 0.1 mM EGTA, 1 mM MgCl_2_, 10 mM HEPES, 2 mM phosphate, 0.3% BSA, 0.5 mM ADP (Sigma-Aldrich), 5 mM pyruvate, 10 mM succinate and 10 mM glucose, followed by immediate addition of 5 units of hexokinase (Sigma-Aldrich) and incubation at 37 °C for 30 min. The reaction was terminated by addition of 5% ice cold trichloroacetic acid (TCA) and the amount of inorganic phosphate was measured spectrophotometrically. A 0 min sample was also assayed for inorganic phosphate content where hexokinase addition was immediately followed by treatment with 5% ice cold TCA. Glucose and hexokinase in the reaction mixture acted as a trap for ATP to maintain the level of ADP in the system and also to prevent the release of free inorganic phosphate from ATP by the action of various phosphatases.

### ATP synthesis

ATP content was measured in aliquots of mitochondrial suspension by a colorimetric assay method using the phosphorylation of glycerol to generate a quantifiable product at 570 nm using a commercial ATP assay kit (ab83355, Abcam).

### Measurement of mitochondrial transmembrane potential

Murine colon: aliquots of mitochondrial suspensions were incubated at 37 °C for 30 min in isotonic buffer A containing 10 mM pyruvate, 10 mM succinate and 1 mM ADP with 5 *μ*M JC-1 (5,5′,6,6′-tetrachloro-1,1′,3,3′- tetraethyl benzimidazolylcarbocyanine iodide, CS0760, Sigma-Aldrich). After incubation, the dyed mitochondria were collected by centrifugation, washed with isotonic buffer A to remove excess dye and resuspended in the same buffer in appropriate dilution, followed by measurement of fluorescence intensity (*λ*ex 490 nm, *λ*em 590 nm). *In vitro* mucosal surface: Mitochondrial transmembrane potential in intact HT29 MTX-E12 cells was measured using a tetramethylrhodamine ethyl ester (TMRE) mitochondrial membrane potential assay kit (ab113852, Abcam) following the manufacturer’s instructions.

### Measurement of citrate synthase activity

The activity was determined spectrophotometrically at 30 °C according to the method of Srere (1969)^63^. The assay medium consisted of 0.1 M tris-HCl (pH-8.5), 0.1 mM 5-dithiobis-2- nitrobenzoic acid (DTNB), acetyl CoA, 500 μM oxaloacetic acid, and a mitochondrial suspension (10 *μ*g protein) in a total volume of 1 ml. As citrate synthase irreversibly catalyzes the reaction CoA-SH + DTNB → TNB + CoA-S-S-TNB, the readout product used was thionitrobenzoic acid (TNB) which can be quantified by absorption at 412 nm. One unit of enzyme is defined as 1 μmol of oxaloacetate utilized/min per mg of protein.

### Measurement of total nitrite release

Nitrite levels were determined in the cell free supernatant obtained from cell and tissue homogenate (see mitochondria isolation section). Nitrate reductase (ab156629, Abcam) was used to convert nitrates to nitrites and the rest of the procedure was adapted from the Griess reagent kit (G-7921, Life Technologies).

### Protein concentration

Estimated after solubilizing the samples in 1% SDS following the method of Lowry^64^.

### Statistical analysis

All tests were performed using Prism (GraphPad Software, version 3·0) or SPSS statistics 18 (IBM). Values are expressed as mean ± S.E.M. Comparison of data between control and infected at a specific time-point was made using the unpaired *t* test. Differences were considered significantly different if *P* was <0.05. One-way analysis of variance (ANOVA) with Student Newman-Keuls Multiple Comparison test was used to compare data for more than 2 experimental groups. Normality was confirmed using the Kolmogorov-Smirnoff test, and homoscedasticity was confirmed using the Bartlett’s test. Only one data set failed the test (Fig. 2D), but passed these tests after Log10 transformation. For Figs 5 and 9, the number of n in each treatment group was too small (n = 3) to perform these tests, however, overall the data followed a normal distribution, and in experiments with several treatments and balanced data, heterogeneous variances do not noticeably increase the risk of Type I error^65^. All aspects considered, we decided One-Way ANOVA followed by post hoc testing using Student Newman-Keuls test was the most appropriate way to treat the data since One-Way ANOVA is a robust statistical test if sample sizes are similar. The Pearson product-moment correlation coefficient was used for analyzing correlations.

## Additional Information

**How to cite this article**: Maiti, A. K. *et al*. IL-4 Protects the Mitochondria Against TNFa and IFNγ Induced Insult During Clearance of Infection with *Citrobacter rodentium* and *Escherichia coli*. *Sci. Rep*. **5**, 15434; doi: 10.1038/srep15434 (2015).

## Figures and Tables

**Table 1:** Changes in mRNA level of cytokines in wildtype and IFNγ knockout mice infected with *C. rodentium*.

Cytokine	Fold changes in mRNA levels			
	Day 10 wt	Day14 wt	Day19 wt	Day10 IFNγ−/−
IFNγ	8.3	13.7	72.5	NA
TNFα	1.5	4.7	11.4	2.5
IL-12	2.7	22.7	42.9	2.2
IL-4	1.1	6.9	4.1	2.8
IL-6	2	2.2	6.5	20.8

mRNA from two sets of two mice in each group were pooled for the time points of day 0 and day 10 (i.e. data are representative of four mice in each group), whereas the time points day 14 and 19 contained mRNA pooled from three mice in each group. Data are presented as fold change compared to uninfected control mice of the same genotype. Data were normalized by the RT2 Profiler PCR Array data analysis software (QIAGEN) using the housekeeping genes Gusb, Hprt1, Hsp90ab1, Gapdh and Actb. Fold changes ≥2,5 were accepted as upregulation.

## References

[b1] Barnett F. D., Abul-Milh M., Huesca M. & Lingwood C. A. Enterohemorrhagic Escherichia coli induces apoptosis which augments bacterial binding and phosphatidylethanolamine exposure on the plasma membrane outer leaflet. Infect. Immun. 68(6), 3108–3115 (20l0). 10816451 10.1128/iai.68.6.3108-3115.2000 PMC97539

[b2] Deng W., Li Y., Vallance B. A. & Finlay B. B. Locus of enterocyte effacement from Citrobacter rodentium: sequence analysis and evidence for horizontal transfer among attaching and effacing pathogens. Infect. Immun. 69(10), 6323–6335 (2001). 11553577 10.1128/IAI.69.10.6323-6335.2001 PMC98768

[b3] Frankel G. . Intimin from enteropathogenic Escherichia coli restores murine virulence to a Citrobacter rodentium eaeA mutant: induction of an immunoglobulin A response to intimin and EspB. Infect. Immun. 64, 5315–5325 (1996). 8945583 10.1128/iai.64.12.5315-5325.1996 PMC174525

[b4] Luperchio S. A. & Schauer D. B. Molecular pathogenesis of Citrobacter rodentium and transmissible murine colonic hyperplasia. Microbes Infect. 3, 333–340 (2001). 11334751 10.1016/s1286-4579(01)01387-9

[b5] Vallance B. A., Deng W., Jacobson K. & Finlay B. B. Host susceptibility to the attaching and effacing bacterial pathogen Citrobacter rodentium. Infect. Immun. 71, 3443–3453 (2003). 12761129 10.1128/IAI.71.6.3443-3453.2003 PMC155702

[b6] Griffin P. M., Olmstead L. & Petras R. E. Escherichia coli O157:H7-associated colitis. A clinical and histological study of 11 cases. Gastroenterology. 99(1), 142–149 (1990). 2188868 10.1016/0016-5085(90)91241-w

[b7] Crane J. K., Majumdar S. & Pickhardt D. F. 3rd. Host cell death due to enteropathogenic Escherichia coli has features of apoptosis. Infect Immun. 67(5), 2575–2584 (1999). 10225923 10.1128/iai.67.5.2575-2584.1999 PMC116006

[b8] Kroemer G., Zamzami N. & Susin S. A. Mitochondrial control of apoptosis. Immunology today 18, 44–51 (1997). 9018974 10.1016/s0167-5699(97)80014-x

[b9] Desagher S. & Martinou J. Mitochondria as the central control point of apoptosis. Trends Cell Biol. 10(9), 369–377 (2000). 10932094 10.1016/s0962-8924(00)01803-1

[b10] Dean P., Maresca M. & Kenny B. EPEC’s weapons of mass subversion. Curr. Opin. Microbiol. 8, 28–34 (2005). 15694854 10.1016/j.mib.2004.12.010

[b11] Deng W. . Dissecting virulence: systematic and functional analyses of a pathogenicity island. Proc. Natl. Acad. Sci USA. 101(10), 3597–3602 (2004). 14988506 10.1073/pnas.0400326101 PMC373508

[b12] Schauer D. B. & Falkow S. Attaching and effacing locus of a Citrobacter freundii biotype that causes transmissible murine colonic hyperplasia. Infect. Immun. 61(6), 2486–2492 (1993). 8500884 10.1128/iai.61.6.2486-2492.1993 PMC280873

[b13] Ma C. . Citrobacter rodentium infection causes both mitochondrial dysfunction and intestinal epithelial barrier disruption *in vivo*: role of mitochondrial associated protein (Map). Cell Microbiol. 8(10), 1669–1686 (2006). 16759225 10.1111/j.1462-5822.2006.00741.x

[b14] Mundy R., MacDonald T., Dougan G., Frankel G. & Wiles S. Citrobacter rodentium of mice and man. Cell Microbiol. 7(12), 1697–1706 (2005). 16309456 10.1111/j.1462-5822.2005.00625.x

[b15] Nougayrède J. P. & Donnenberg M. Enteropathogenic Escherichia coli EspF is targeted to mitochondria and is required to initiate the mitochondrial death pathway. Cell Microbiol. 6(11), 1097–1111 (2004). 15469437 10.1111/j.1462-5822.2004.00421.x

[b16] Nagai T., Abe A. & Sasakawa C. Targeting of enteropathogenic Escherichia coli EspF to host mitochondria is essential for bacterial pathogenesis: critical role of the 16th leucine residue in EspF. J. Biol. Chem. 280, 2998–3011 (2005). 15533930 10.1074/jbc.M411550200

[b17] Lopez-Armada M. J., Riveiro-Naveira R. R., Vaamonde-Garcia C. & Valcarcel-Ares M. N. Mitochondrial dysfunction and the inflammatory response. Mitochondrion 13, 106–118 (2013). 23333405 10.1016/j.mito.2013.01.003

[b18] Navabi N., McGuckin M. & Lindén S. K. Gastrointestinal cell lines form polarized epithelia with an adherent mucus layer when cultured in semi-wet interfaces with mechanical stimulation. PLoS One. 8(7), 68761 (2013). 10.1371/journal.pone.0068761 PMC3712011 23869232

[b19] Gustafsson J. K. . Dynamic changes in mucus thickness and ion secretion during Citrobacter rodentium infection and clearance. PloS one 8(12), e84430 10.1371/journal.pone.0084430 (2013). 24386378 PMC3875541

[b20] Srinivasula S. M. & Ashwell J. D. IAPs: what’s in a name? Mol. cell 30, 123–135 (2008). 18439892 10.1016/j.molcel.2008.03.008 PMC2677451

[b21] Boatright K. M. & Salvesen G. S. Mechanisms of caspase activation. Curr. Opin. Cell Biol. 15, 725–731 (2003). 14644197 10.1016/j.ceb.2003.10.009

[b22] Florey H. W.. Electron microscopic observations on goblet cells of the rat’s colon. Q. J. Exp. Physiol. Cogn. Med. Sci. 45 329–336 (1960). 13700347 10.1113/expphysiol.1960.sp001487

[b23] Ding W. X. & Yin X. M. Mitophagy: mechanisms, pathophysiological roles, and analysis. Biol. Chem. 393(7), 547–64 (2012). 22944659 10.1515/hsz-2012-0119 PMC3630798

[b24] Higgins L. M., Frankel G., Douce G., Dougan G. & MacDonald T. T. *Citrobacter rodentium* infection in mice elicits a mucosal Th1 cytokine response and lesions similar to those in murine inflammatory bowel disease. Infect Immun. 67(6), 3031–3039 (1999). 10338516 10.1128/iai.67.6.3031-3039.1999 PMC96617

[b25] Smith R. A. & Murphy M. P. Animal and human studies with the mitochondria-targeted antioxidant MitoQ. Ann N Y Acad. Sci. 1201, 96–103 (2010). 20649545 10.1111/j.1749-6632.2010.05627.x

[b26] Taylor R. W., Birch-Machin M. A., Bartlett K. & Turnbull D. M. Succinate-cytochrome c reductase: assessment of its value in the investigation of defects of the respiratory chain. Biochimica et biophysica Acta 1181, 261–265 (1993). 8391327 10.1016/0925-4439(93)90030-5

[b27] Muller A. . Targeting of the pro-apoptotic VDAC-like porin (PorB) of Neisseria gonorrhoeae to mitochondria of infected cells. The EMBO journal 19, 5332–5343 (2000). 11032801 10.1093/emboj/19.20.5332 PMC314008

[b28] Massari P., King C., Ho A. Y. & Wetzler L. M. Neisserial PorB is translocated to the mitochondria of HeLa cells infected with Neisseria meningitidis and protects cells from apoptosis. Cell Microbiol. 5(2), 99–109 (2003). 12580946 10.1046/j.1462-5822.2003.00257.x

[b29] Galmiche A. . The N-terminal 34 kDa fragment of Helicobacter pylori vacuolating cytotoxin targets mitochondria and induces cytochrome c release. The EMBO journal 19, 6361–6370 (2000). 11101509 10.1093/emboj/19.23.6361 PMC305856

[b30] Hernandez L. D., Pypaert M., Flavell R. A. & Galan J. E. A Salmonella protein causes macrophage cell death by inducing autophagy. J. Cell Biol. 163, 1123–1131 (2003). 14662750 10.1083/jcb.200309161 PMC2173598

[b31] Ohteki T. . Interleukin 12-dependent interferon gamma production by CD8alpha + lymphoid dendritic cells. J. Exp. Med 189, 1981–1986 (1999). 10377194 10.1084/jem.189.12.1981 PMC2192968

[b32] Shiomi H. . Gamma interferon produced by antigen-specific CD4 + T cells regulates the mucosal immune responses to Citrobacter rodentium infection. Infect. Immun. 78, 2653–2666 (2010). 20351140 10.1128/IAI.01343-09 PMC2876554

[b33] Dalton D. K. . Multiple defects of immune cell function in mice with disrupted interferon-gamma genes. Science 259, 1739–1742 (1993). 8456300 10.1126/science.8456300

[b34] Schroder K., Hertzog P. J., Ravasi T. & Hume D. A. Interferon-gamma: an overview of signals, mechanisms and functions. Journal of leukocyte biology 75, 163–189 (2004). 14525967 10.1189/jlb.0603252

[b35] Busquets S. . Tumour necrosis factor-alpha uncouples respiration in isolated rat mitochondria. Cytokine 22, 1–4 (2003). 12946099 10.1016/s1043-4666(03)00098-x

[b36] Lancaster J. R. Jr., Laster S. M. & Gooding L. R. Inhibition of target cell mitochondrial electron transfer by tumor necrosis factor. FEBS letters 248, 169–174 (1989). 2721674 10.1016/0014-5793(89)80454-5

[b37] Ledgerwood E. C. . Tumor necrosis factor is delivered to mitochondria where a tumor necrosis factor-binding protein is localized. Lab. Invest. 78(12), 1583–1589 (1998). 9881958

[b38] Geng Y., Hansson G. & Holme E. Interferon-gamma and tumor necrosis factor synergize to induce nitric oxide production and inhibit mitochondrial respiration in vascular smooth muscle cells. Circ. Res. 71(5), 1268–1276 (1992). 1394884 10.1161/01.res.71.5.1268

[b39] Kamachi M. . Regulation of apoptotic cell death by cytokines in a human salivary gland cell line: distinct and synergistic mechanisms in apoptosis induced by tumor necrosis factor alpha and interferon gamma. J. Lab. Clin. Med. 139(1), 13–19 (2002). 11873240 10.1067/mlc.2002.120648

[b40] Lemaire C., Andr Au, K., Fraisse C. S., Adam A. & Souvannavong V. IL-4 inhibits apoptosis and prevents mitochondrial damage without inducing the switch to necrosis observed with caspase inhibitors. Cell Death Differ. 6(8), 813–820 (1999). 10467356 10.1038/sj.cdd.4400556

[b41] Dann S. M. . IL-6-dependent mucosal protection prevents establishment of a microbial niche for attaching/effacing lesion-forming enteric bacterial pathogens. J. Immunol. 180, 6816–6826 (2008). 18453602 10.4049/jimmunol.180.10.6816 PMC2696063

[b42] James A. M., Cocheme H., Smith R. A. & Murphy M. P. Interactions of mitochondria-targeted and untargeted ubiquinones with the mitochondrial respiratory chain and reactive oxygen species. Implications for the use of exogenous ubiquinones as therapies and experimental tools. J Biol. Chem. 280(22), 21295–21312 (2005). 15788391 10.1074/jbc.M501527200

[b43] Gobert A. P. . Protective role of arginase in a mouse model of colitis. J Immunol. 173(3), 2109–17 (2004). 15265947 10.4049/jimmunol.173.3.2109

[b44] Kowaltowski A. J. & Vercesi A. E. Mitochondrial damage induced by conditions of oxidative stress. Free Radic. Biol. Med. 26, 463–471 (1999). 9895239 10.1016/s0891-5849(98)00216-0

[b45] Schon E. A., DiMauro S. & Hirano M. Human mitochondrial DNA: roles of inherited and somatic mutations. Nat.Rev. Genet. 13, 878–890 (2012). 23154810 10.1038/nrg3275 PMC3959762

[b46] Wang A. . Targeting mitochondria-derived reactive oxygen species to reduce epithelial barrier dysfunction and colitis. Am. J. Pathol. 184, 2516–2527 (2014). 25034594 10.1016/j.ajpath.2014.05.019 PMC4188172

[b47] Dashdorj A. . Mitochondria-targeted antioxidant MitoQ ameliorates experimental mouse colitis by suppressing NLRP3 inflammasome-mediated inflammatory cytokines. BMC medicine 11, 178 (2013). 23915129 10.1186/1741-7015-11-178 PMC3750576

[b48] Zund G., Madara J. L., Dzus A. L., Awtrey C. S. & Colgan S. P. Interleukin-4 and interleukin-13 differentially regulate epithelial chloride secretion. J.Biol.Chem. 271, 7460–7464 (1996). 8631774 10.1074/jbc.271.13.7460

[b49] Smirnova M. G., Kiselev S. L., Birchall J. P. & Pearson J. P. Up-regulation of mucin secretion in HT29-MTX cells by the pro-inflammatory cytokines tumor necrosis factor-alpha and interleukin-6. Eur Cytokine Netw 12, 119–125 (2001). 11282555

[b50] Hiscox S., Hallett M. B., Puntis M. C. & Jiang W. G. Inhibition of cancer cell motility and invasion by interleukin-12. Clin. Exp. Metastasis 13, 396–404 (1995). 7641424 10.1007/BF00121916

[b51] Adams R. B., Planchon S. M. & Roche J. K. IFN-gamma modulation of epithelial barrier function. Time course, reversibility, and site of cytokine binding. J. Immunol. 150, 2356–2363 (1993). 8450217

[b52] Harrop C. A., Gore R. B., Evans C. M., Thornton D. J. & Herrick S. E. TGF-beta(2) decreases baseline and IL-13-stimulated mucin production by primary human bronchial epithelial cells. Exp. Lung Res. 39, 39–47 (2013). 23249391 10.3109/01902148.2012.748854

[b53] Treede I. . TNF-alpha-induced up-regulation of pro-inflammatory cytokines is reduced by phosphatidylcholine in intestinal epithelial cells. BMC gastroenterology 9, 53 (2009). 19594939 10.1186/1471-230X-9-53 PMC2714528

[b54] Tarant J. M. Blood cytokines as biomarkers of *in vivo* toxicity in preclinical safety assessment: considerations for their use. Toxicol Sci. 117(1), 4–16 (2010). 20447938 10.1093/toxsci/kfq134 PMC2923281

[b55] Miyakawa N. . Prolonged circulation half-life of interferon gamma activity by gene delivery of interferon gamma-serum albumin fusion protein in mice. J. Pharm. Sci. 100, 2350–2357 (2011). 21246562 10.1002/jps.22473

[b56] Finkelman F. D. & Morris S. C. Development of an assay to measure *in vivo* cytokine production in the mouse. Int. Immunol. 11, 1811–1818 (1999). 10545485 10.1093/intimm/11.11.1811

[b57] Flach C. F., Ostberg A. K., Nilsson A. T., Malefyt Rde W. & Raghavan S. Proinflammatory cytokine gene expression in the stomach correlates with vaccine-induced protection against Helicobacter pylori infection in mice: an important role for interleukin-17 during the effector phase. Infect Immun 79, 879–886 (2011). 21078851 10.1128/IAI.00756-10 PMC3028834

[b58] Modica-Napolitano J. S., Steele G. D. Jr. & Chen L. B. Aberrant mitochondria in two human colon carcinoma cell lines. Cancer Res. 49, 3369–3373 (1989). 2720690

[b59] Hatefi Y. Preparation and properties of NADH: ubiquinone oxidoreductase (complexI), EC 1.6.5.3. Methods Enzymol. 53, 11–14 (1978). 713832 10.1016/s0076-6879(78)53006-1

[b60] Wharton D. C. & Tzagoloff A. Cytochrome oxidase from beef heart mitochondria. Methods Enzymol. 10, 245–250 (1967).

[b61] Clark J. B., Bates T. E., Boakye P., Kuimov A. & Land J. M. Investigation of mitochondrial defects in brain and skeletal muscle. In: Neurochemistry: A Practical Approach (Turner A. J. & Bachelard H. S. Eds.) pp. 151–174, Oxford University Press, Oxford (1997).

[b62] Hinkle P. C. Oxygen proton and phosphate fluxes and stoichiometries. in: Bioenergetics, A Practical Approach (Brown G. C. & Cooper C. E. Eds.) pp 1–15 IRL Press, Oxford (1995).

[b63] Srere P. A. Citrate Synthase. Methods Enzymol. 13, 3–11 (1969).

[b64] Lowry O. H., Rosebrough N. J., Farr A. L. & Randall R. J. Protein measurement with the Folin phenol reagent. J. Biol. Chem. 193, 265–275 (1951). 14907713

[b65] Underwood A. J. Environmental decision-making and the precautionary principle: What does this principle mean in environmental sampling practice? Landscape Urban Plan 37, 137–146 (1997).

